# Applications of Exosomal miRNAs from Mesenchymal Stem Cells as Skin Boosters

**DOI:** 10.3390/biom14040459

**Published:** 2024-04-09

**Authors:** Jinmei Zheng, Beibei Yang, Siqi Liu, Zhenfeng Xu, Zhimeng Ding, Miaohua Mo

**Affiliations:** 1 Department of Biotechnology, Guangdong Medical University, Dongguan 523808, China; zjmei@gdmu.edu.cn (J.Z.); yangbb@gdmu.edu.cn (B.Y.); siqiliu@gdmu.edu.cn (S.L.); xuzhenfeng@gdmu.edu.cn (Z.X.); dzm13590734690@gdmu.edu.cn (Z.D.); 2 Institute of Aging Research, Guangdong Provincial Key Laboratory of Medical Molecular Diagnostics, Guangdong Medical University, Dongguan 523808, China

**Keywords:** mesenchymal stem cells, exosomes, miRNAs, skin regeneration and rejuvenation, bioengineering approaches

## Abstract

The skin is the outer layer of the human body, and it is crucial in defending against injuries and damage. The regenerative capacity of aging and damaged skin caused by exposure to external stimuli is significantly impaired. Currently, the rise in average life expectancy and the modern population’s aesthetic standards have sparked a desire for stem-cell-based therapies that can address skin health conditions. In recent years, mesenchymal stem cells (MSCs) as therapeutic agents have provided a promising and effective alternative for managing skin regeneration and rejuvenation, attributing to their healing capacities that can be applied to damaged and aged skin. However, it has been established that the therapeutic effects of MSC may be primarily mediated by paracrine mechanisms, particularly the release of exosomes (Exos). Exosomes are nanoscale extracellular vesicles (EVs) that have lipid bilayer and membrane structures and can be naturally released by different types of cells. They influence the physiological and pathological processes of recipient cells by transferring a variety of bioactive molecules, including lipids, proteins, and nucleic acids such as messenger RNAs (mRNAs) and microRNAs (miRNAs) between cells, thus playing an important role in intercellular communication and activating signaling pathways in target cells. Among them, miRNAs, a type of endogenous regulatory non-coding RNA, are often incorporated into exosomes as important signaling molecules regulating protein biosynthesis. Emerging evidence suggests that exosomal miRNAs from MSC play a key role in skin regeneration and rejuvenation by targeting multiple genes and regulating various biological processes, such as participating in inflammatory responses, cell migration, proliferation, and apoptosis. In this review, we summarize the recent studies and observations on how MSC-derived exosomal miRNAs contribute to the regeneration and rejuvenation of skin tissue, with particular attention to the applications of bioengineering methods for manipulating the miRNA content of exosome cargo to improve their therapeutic potential. This review can provide new clues for the diagnosis and treatment of skin damage and aging, as well as assist investigators in exploring innovative therapeutic strategies for treating a multitude of skin problems with the aim of delaying skin aging, promoting skin regeneration, and maintaining healthy skin.

## 1. Introduction

Skin, as the largest and outermost organ, functions as the first line of defense against injury and infection by providing a physical barrier and immune protection. Wounds and aging are two consequences of skin exposure to injuries. Wounds and skin damage can be induced by trauma, lacerations, abrasions, burns, various surgeries, or an underlying disease such as diabetes or vascular disease. In addition, everyone experiences aging from birth, and skin aging is a natural process and a direct reflection of the body’s aging. Skin aging can be caused by both intrinsic and extrinsic factors, and both intrinsic and extrinsic aging can result in a decrease in the structural integrity of the skin and a loss of physiological function [1,2]. Furthermore, the ability of aging skin to regenerate is significantly impaired due to the constant internal and external stress it is subjected to. Currently, wounds have emerged as a major public health concern that may have a tremendous impact on health-related quality of life and the cost of the health care system [3,4,5]. Additionally, delaying skin aging is crucial in maintaining people’s normal physiological functions of the skin; thus, the therapeutic strategy for preventing or delaying skin aging has attracted great attention, which is necessary for developing skin care products and delaying the risk of skin aging. In recent years, due to the continuous improvement of material life and the extension of life expectancy, the level of modern people’s awareness of skin health has improved. There is an increasing concern about accidents, burns, diseases, and medical treatments that affect the skin. At the same time, the application of mesenchymal stem cells in the treatment of skin regeneration is also becoming more and more widespread. For those reasons, tissue regeneration and rejuvenation represent fields of interest in clinical practice. Regeneration and rejuvenation therapies, which focus on the repair and regeneration of damaged skin, as well as the prevention and reversal of skin aging, are in high demand in our society.

Mesenchymal stem cells are pluripotent stem cells that originate from adult stem cells and have the ability to self-renew and self-repair, as well as the capacity to differentiate into multiple functional cells under certain conditions [6,7,8,9]. Primary MSC can be obtained from diverse sources, including placental tissue, umbilical cord tissue, bone marrow, adipose tissue, umbilical cord blood, and other tissues [6,7,8,9]. Recently, MSC have been recognized as a therapeutic agent for skin regeneration and rejuvenation due to their superior characteristics that can be applied to damaged and aged skin, such as their ease of isolation and expansion, multi-potential, anti-inflammatory, anti-apoptotic, and immunomodulatory properties, and their capability to replace damaged cells and restore tissue function [6,7,8,9]. Nevertheless, increasing studies have shown that implantation of MSC in vivo significantly promotes tissue repair or delays senescence, but it is surprising that the implanted cells are only transiently detected within the tissues; these findings suggest that MSCs perform their functions through a paracrine signal transduction mechanism, particularly those involving extracellular vesicles [10,11].

Extracellular vesicles are a complex mixture of membrane-bound vesicles released from most cells, and they can be divided into three different subgroups according to their size and biogenesis: exosomes (Exos, 50–150 nm), microvesicles (MVs, 100–1000 nm), and apoptotic bodies (ABs, 500–5000 nm) [12,13]. Exosomes are nanosized endocytic vesicles originating from multi-vesicular bodies, which are usually secreted into the extracellular matrix by fusion with the cytomembrane [12,14]. According to recent studies, exosomes are the most important structures involved in paracellular secretion, which influence a series of pathophysiological processes by transporting their contents into target cells and participating in cell-to-cell communication [14]. Additionally, as an alternative to cell-free therapy, exosomes have several advantages over stem cell transplantation, including stability, low toxicity, biocompatibility, immune rejection and tumorigenesis risk avoidance, and skilled exchange of molecular goods, making them an ideal candidate for tissue engineering and regenerative medicine [7,8,15,16]. Based on this, much attention has been focused on the study of exosomes, especially mesenchymal-stem-cells-derived exosomes (MSC-Exos). Indeed, MSC-Exos exert their effects mainly by releasing a variety of bioactive molecules [17,18,19]. Exosomes, depending on their parental origin and microenvironment, carry a large diversity of cargos, including mRNAs, long non-coding RNAs (lncRNAs), miRNAs, proteins, and lipids, and play key roles in intercellular communication and information transmission by transporting and delivering their biologically active molecules to neighboring or distant cells through a unique mechanism [12,13,14]. In particular, exosomes can be loaded with miRNAs that can be transferred to nearby cells or targeted cells and then internalized by recipient cells, resulting in an alteration of cellular functions [14,20]. MiRNAs are a type of endogenous, non-protein-coding, short RNA molecules that are between 19 and 22 nucleotides long. They are significant post-transcriptional gene regulators that can either fully or partially regulate a number of mRNAs, which prevents the synthesis of proteins [20,21,22].

Skin damage and aging can occur when the skin is constantly exposed to various external stimuli. In dermatology, there have been many reported cases in which MSC-secreted exosomes have the potential for skin regeneration and rejuvenation, such as tissue damage repair, chronic wound healing, inhibition of wound scar formation, and anti-aging [8,23,24,25,26,27,28]. MSC-Exos play a role in skin regeneration by increasing cell proliferation and neovascularization and decreasing inflammation in skin injury lesions [26,27,28]. Simultaneously, skin rejuvenation is facilitated by MSC-Exos through the production of collagen and elastic fibers, inhibition of metalloproteinase activation, and enhancement of protection against ultraviolet B (UVB)-radiation-induced aging [23,24,25]. Therefore, the use of exosomes derived from MSC for skin regeneration and rejuvenation is a novel treatment method that has received much attention. Recently, increasing evidence has demonstrated that MSC-Exos carry out critical functions in skin regeneration and rejuvenation by transferring their cargo miRNAs that function as vital players in regulating a variety of biological processes, including angiogenesis, the regression of inflammation, the proliferation and migration of skin cells, as well as the production of collagen and elastic fibers [8,23,26,29,30,31].

In this review, we systematically summarize the latest applications of miRNAs derived from MSC-Exos in improving diseased and aging skin, as well as the intracellular signaling pathways mediated by miRNAs. Additionally, we emphasize the applications of bioengineering methods for manipulating the miRNA content of exosome cargo to improve their therapeutic potential. This review can provide a clear understanding for future clinical diagnosis and treatment of skin damage and aging, as well as assist investigators in exploring new therapeutic strategies and developing skin care products for treating a multitude of skin problems, which can delay the risk of skin aging, promote skin regeneration, and maintain healthy skin.

## 2. Skin Regeneration and Rejuvenation-Associated miRNAs

Recent research indicates that exosomal miRNAs from MSC play a critical role in skin regeneration and rejuvenation, including wound healing, reducing skin scar formation, skin anti-aging, and hair regrowth. The relevant studies are summarized in Table 1.

**Figure 1:**
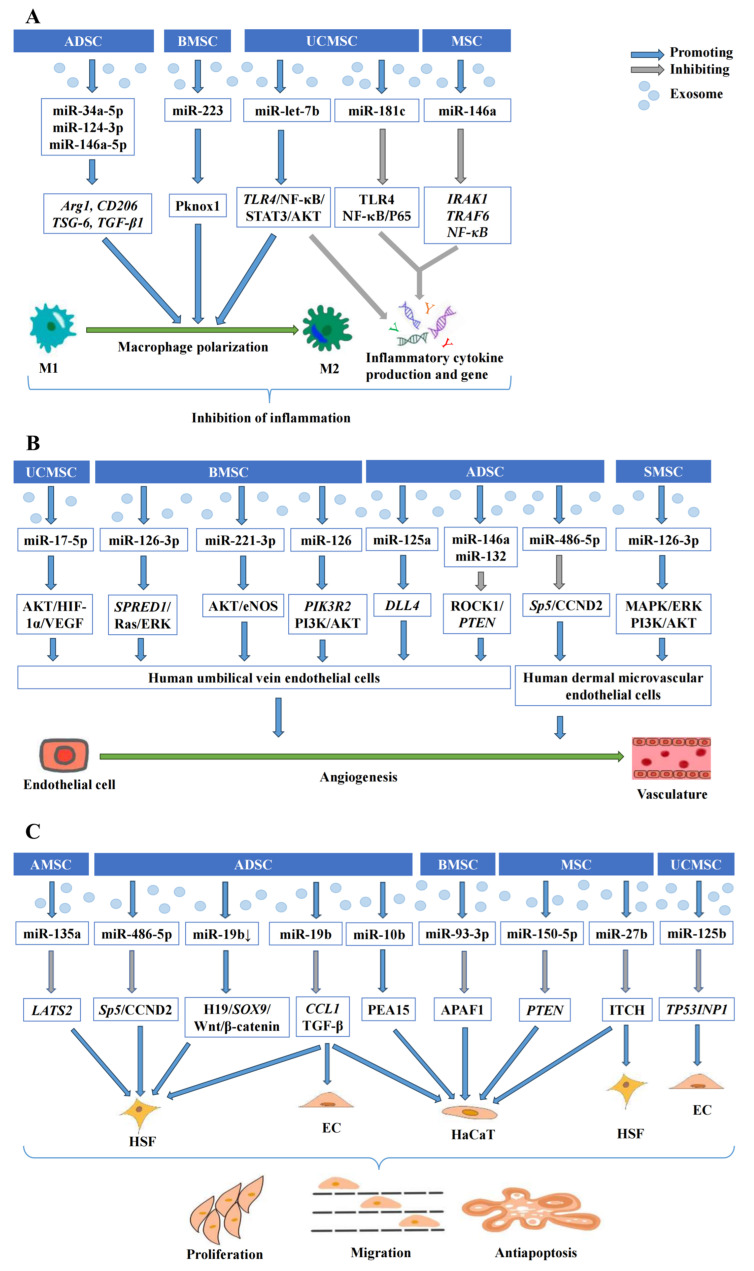
Multiple regulatory mechanisms of MSC-Exos on wound healing are mediated by exosome miRNAs. MSC-Exos are capable of promoting wound healing through a variety of regulatory mechanisms mediated by exosome-shuttling miRNAs. (**A**) MSC-Exos exert wound healing effects by inhibiting the production of inflammatory factors and inflammatory genes and promoting the polarization of M1 macrophages to M2, mediated by multiple exosomal miRNA pathways. (**B**) MSC-Exos promote wound healing via exosomal miRNA-pathway-mediated angiogenesis of human umbilical vein endothelial cells and human dermal microvascular endothelial cells. (**C**) MSC-Exos enhance wound healing through miRNA-mediated pro-proliferative, pro-migratory, and anti-apoptotic effects on a variety of skin cells, including human skin fibroblasts (HSFs), endothelial cells (ECs), and human keratinocytes (HaCaT).

Mesenchymal-stem-cell-exosomes-derived miRNAs are involved in multiple processes of wound healing, including regulating inflammation responses, promoting angiogenesis, and modulating cell proliferation, migration, and apoptosis (Figure 1) [65,66].

### 2.1. Wound Healing

#### 2.1.1. Exosomal miRNAs Derived from MSC Modulate Inflammatory Responses

The stage of inflammation is crucial for skin regeneration [67]. However, the persistence of inflammation is not conducive to wound healing, and the degree of inflammation has a significant impact on the effect of wound healing [65,67]. The initiation and disappearance of inflammatory reactions are the main parts of skin regeneration and also the key to determining the quality and time of healing. Neutrophils and macrophages are the two primary types of cells involved in the inflammatory response.

It is now clear that miRNA-mediated inflammatory responses are mainly associated with two pathways: the Toll-like receptor (TLR) pathway and the nuclear transcription factor-kappa B (NF-κB) pathway [31]. Studies have shown that miR-181c plays an important role in immune cell metabolism and the control of inflammation by fine-tuning immune function. The umbilical cord mesenchymal-stem-cells-derived exosomes (UCMSC-Exos) carrying miR-181c have a more potent effect on alleviating excessive inflammation in burn rats by inhibiting the TLR4 signaling pathway, which in turn leads to a decrease in the activation of NF-κB/p65, a crucial mediator in controlling the production of inflammatory mediators [32]. MiR-146a is one of the important regulatory molecules in the inflammatory response. The inflammatory response is frequently triggered by pro-inflammatory stimuli such as interleukin-1b (IL-1b), tumor necrosis factor-α (TNF-α), and TLR. The pro-inflammatory genes IL-1 receptor-associated kinase 1 (*IRAK1*) and TNF receptor-associated factor 6 (*TRAF6*) are crucial adapter molecules that promote activation of the NF-κB pathway by Toll-like receptors and the interleukin 1 signaling pathway [33,34]. MiR-146a, which is produced from exosomes of mouse bone marrow mesenchymal stem cells (BMSCs), enhances wound healing by suppressing the expression of pro-inflammatory genes *IRAK1* and *TRAF6* [33,34].

Macrophages are important effector cells that have multiple phenotypes and functions in wound healing. Numerous investigations have demonstrated the advantageous effects of macrophage polarization toward macrophage 2 (M2) on wound healing [35]. Inflammation shifts from the inflammatory phase to the tissue repair phase when the inflammatory phenotype macrophage1 (M1) switches to the wound healing/fibrosis-promoting phenotype M2 [66]. Overall, wound healing can be improved by promoting macrophage polarization from the pro-inflammatory phenotype M1 to the anti-inflammatory M2 phenotype. A study reports that miR-223 shuttled by BMSC-derived exosomes accelerates wound healing by targeting the Pknox1 protein to regulate macrophage polarization, shifting macrophages from an M1 phenotype to an M2 phenotype [31,35]. Another study has shown that exosomes secreted by adipose mesenchymal stem cells (ADSCs) are highly expressive of miR-34a-5p, miR-124-3p, and miR-146a-5p, which are important miRNAs associated with the M2 phenotype of macrophages and can induce M2-type macrophage polarization by targeting a variety of transcription factors and proteins, including arginase 1 (*Arg1*), *CD206*, tumor necrosis factor-inducible gene 6 (*TSG-6*), and transforming growth factor beta 1 (*TGF-β1*), thereby reducing inflammation and enhancing wound healing [37].

Interestingly, pretreatment of MSC with chemical or biological factors may enhance the biological activity of MSC-derived exosomes [39]. For instance, exosomes that are derived from MSC treated with pro-inflammatory cytokines like interferon γ (IFN-γ), TNF-α, and lipopolysaccharide (LPS) can have enhanced immunosuppressive and anti-inflammatory properties and shift macrophages from an M1 to an M2 phenotype by shuttling miRNAs that regulate macrophage polarization [17,68,69]. A more specific example is that exosomal miR-let-7b from umbilical cord mesenchymal stem cells (UCMSCs) pretreated with LPS promotes macrophage activation and polarization towards an M2-like profile via the *TLR4*/NF-κB/signal transducer and activator of transcription 3 (STAT3)/serine/threonine-protein kinase B (AKT) regulatory signaling pathway and increases the expression of anti-inflammatory cytokines in macrophages, which attenuates the inflammatory response and accelerates wound healing in cutaneous wounds [36].

In conclusion, proper wound healing requires the regulation of M1–M2 polarization. At the site of the wound, inflammation clears away harmful pathogens and damaged cells. However, uncontrolled inflammation can cause wounds to heal slowly or not at all. MSC-Exos promote macrophage polarization from the pro-inflammatory phenotype M1 to the anti-inflammatory phenotype M2, thereby improving wound healing. Altering this balance can lead to consequences such as failure to heal wounds or increased tissue fibrosis.

#### 2.1.2. Exosomal miRNAs Derived from MSC Promote Angiogenesis

New vascular complexes must quickly form during wound healing in order to supply enough oxygen and nutrients to the newly formed granulation tissue. Pro-angiogenic chemicals that encourage angiogenesis and aid in wound healing can be released by MSC [7].

The most significant element causing angiogenesis is vascular endothelial growth factor (VEGF), a pro-angiogenic factor that can control endothelial cell migration and differentiation as well as enhance endothelial cell recruitment to promote angiogenesis and endothelialization of wound tissues. Moreover, VEGF can stimulate the induction of signaling axes such as mitogen-activated protein kinase (MAPK), phosphatidylinositol 3-kinase (PI3K)/AKT, which also enhance endothelial cell survival, proliferation, migration and wound angiogenesis [7]. Wei et al. demonstrated that exosome-derived miR-17-5p from UCMSC promoted angiogenesis and accelerated wound healing in diabetes through the AKT/hypoxia inducible factor-1 alpha (HIF-1α)/VEGF pathway [26].

The biological activity of MSC-derived exosomes may be enhanced by preconditioning them with chemical or biological factors, which further potentiate the effects of proangiogenic factors. For example, the research from Yu et al. has found that exosomes isolated from atorvastatin-pretreated BMSC, compared with those from non-pretreated BMSC, exhibit much more excellent abilities in facilitating wound regeneration by promoting the proliferation, migration, tube formation, and VEGF level of endothelial cells and granulation tissue formation via the miR-221-3p-mediated activation of the AKT/endothelial nitric oxide synthase (eNOS) pathway [39]. MiR-126 is regarded as the endothelial-specific microRNA that governs vascular integrity and angiogenesis [70]. By targeting phosphoinositol-3 kinase regulatory subunit 2 (*PIK3R2*), miR-126 generated from BMSC stimulates the PI3K/AKT signaling pathway and encourages the migration and proliferation of human umbilical vein endothelial cells (HUVECs), hence promoting angiogenesis and wound healing [40]. Through the activation of MAPK/extracellular signal-related kinases (ERK) and PI3K/AKT, synovial MSC-derived miR-126-3p encapsulated in hydroxyapatite-chitosan composite hydrogel has been proved to improve wound surface re-epithelialization and accelerate angiogenesis [41].

In addition to enhancing the expression of VEGF, angiogenesis can be promoted by regulating the expression of other factors or signaling pathways [7]. Likewise, IFN-γ-pretreated exosome miR-126-3p promotes the proliferation, migration, and angiogenesis of HUVEC by targeting sprouty-related EVH1 domain-containing protein 1 (*SPRED1*) and the downstream Ras/ERK pathway [42]. MiR-125a, which is released by adipose-mesenchymal-stem-cells-derived exosomes (ADSC-Exos), inhibits delta-like 4 (*DLL4*) and stimulates the production of endothelium tip cells to increase angiogenesis and expedite wound healing [43]. MiR-132 and miR-146a derived from ADSC exosomes up-regulates the expression of angiopoietin1 (*ANGPT1*) and kinase insert domain receptor (*KDR*) (pro-angiogenesis genes) and down-regulates the expression of vasohibin-1 (*VASH1*) and anti-angiogenesis genes thrombosopondin-1 (*THBS1*) through rho associated coiled-coil containing protein kinase 1 (*ROCK1*)/phosphatase and tensin homolog(*PTEN)* signaling pathways to promote endothelial cell angiogenesis [38]. By suppressing *Sp5*/cyclin D2 (CCND2) expression, miR-486-5p released by ADSC-EVs stimulates angiogenesis in human microvascular endothelial cells (HMECs), which mediates the process of skin wound healing [44].

#### 2.1.3. Exosomal miRNAs Derived from MSC Regulate Cell Proliferation, Migration, and Apoptosis

MiRNAs shuttled by MSC exosomes have emerged as important signaling molecules in skin wound healing, affecting skin cell proliferation, migration, and apoptosis. The biological process of wound healing is also crucial for skin repair and regeneration, which mainly involves the proliferation and migration of human keratinocytes, fibroblasts, and endothelial cells [22].

The migration and replication of keratinocytes during the proliferative phase are necessary for wound closure because they drive the re-formation of the epidermis. MiRNAs, which are closely related to the proliferation and migration of keratinocytes, are considered as potential targets for developing novel wound-healing drugs or wound-healing therapies [22]. In Shen et al.’s study, the exosome miR-93-3p secreted by BMSC was observed to promote the migration and proliferation of epithelial HaCaT cells by targeting apoptotic peptidase-activating factor 1 (APAF1), thereby playing a protective role in wound healing [27]. Cheng and his colleagues’ research indicated that MSC-derived exosomal miR-150-5p accelerates skin wound healing via the *PTEN*-mediated PI3K/AKT pathway since miR-150-5p down-regulates *PTEN*, thereby promoting the proliferation and migration of H_2_O_2_-induced HaCaT cells and inhibiting their apoptosis [47]. In a similar study, it was found that miR-10b from ADSC-EVs can enhance skin wound healing by targeting PEA15 to promote cyclin dependent kinase 6 (CDK6) expression, thereby promoting the proliferation and migration of H_2_O_2_-damaged HaCaT cells while inhibiting their apoptosis [49]. MSC-derived EVs harboring miR-27b activate the JUNB/IRE1α axis and enhance the proliferation and migration of HaCaT and HSF cells by inhibiting itchy E3 ubiquitin protein ligase (ITCH) in vitro and in vivo to promote skin wound healing [48].

Fibroblasts are the primary target and effector cells of skin wound healing and also interact with surrounding keratinocytes and collagen during the healing process [45]. By producing growth factors like VEGF, platelet-derived growth factor (PDGF), and other growth factors, fibroblasts encourage the migration, proliferation, and alignment of endothelial cells. By suppressing *Sp5*/CCND2 expression, miR-486-5p released by ADSC-EVs encourages HSF migration and proliferation, which mediates the process of skin wound healing [44]. Exosomal miR-135a generated from human amniotic mesenchymal stem cells (AMSCs) has been shown to down-regulate large tumor suppressor 2 (*LATS2*) levels, which in turn speeds up skin fibroblast migration and facilitates cutaneous wound healing in rats [28]. Qian et al. have demonstrated that overexpression of lncRNA H19 (H19) in ADSC-Exos can down-regulate miR-19b and increase the expression of sex-determining region Y (SRY)-related high-mobility-group box 9 (*SOX9*), which in turn can activate the Wnt/β-catenin pathway and promote the proliferation and migration of HSF, thereby accelerating the regeneration of skin wounds [45]. On the other hand, the work of Cao et al. showed that the ADSC-derived exosome miR-19b targeted the chemokine CC motif ligand 1 (*CCL1*), modulated the TGF-β signaling pathway, accelerated the roles of HaCaT cells and HSF in injury recovery, and also attenuated endothelial cell apoptosis, thereby promoting wound healing [46]. Hypoxic conditions can be applied to enhance the biological properties of MSC-derived exosomes to promote wound healing. To enhance skin wound healing, umbilical cord mesenchymal stem cells were pretreated with hypoxic conditions, and their derived exosome miR-125b targeted the tumor protein p53 inducible nuclear protein 1 (*TP53INP1*) to inhibit *TP53INP1*-mediated apoptosis and stimulate endothelial cell migration and proliferation [50].

### 2.2. Skin Scar

**Figure 2:**
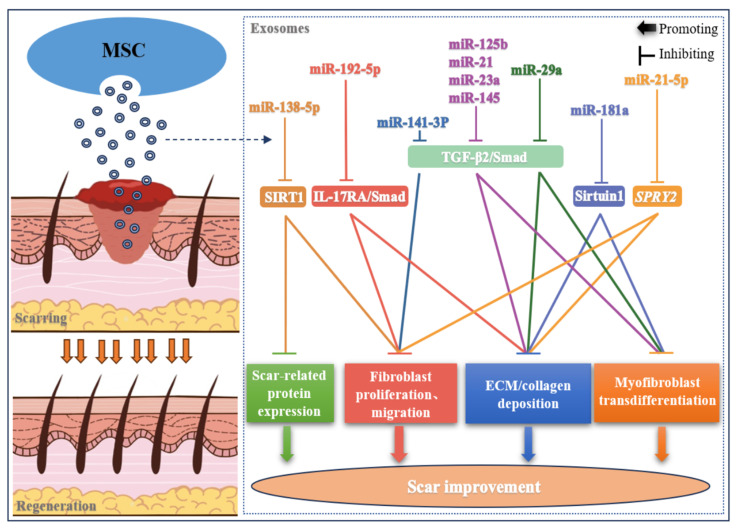
Role and regulatory mechanisms of exosomal miRNAs derived from mesenchymal stem cells in improving skin scar regeneration. Mesenchymal-stem-cell-derived exosomal miRNAs exert their ability to ameliorate scarring by targeting relevant molecules to inhibit the expression of scar-associated proteins, suppress fibroblast proliferation and migration, inhibit the deposition of extracellular matrix (ECM) (e.g., collagen), and prevent the transdifferentiation of myofibroblasts.

Wound healing is a complex process that involves the proper coordination of a range of regulatory factors to allow the repair of damaged tissue and the restoration of normal skin function. In the process of wound healing, the imbalance of any factor at any stage can lead to the formation of skin scars. Scars are areas of fibrous tissue that replace normal skin after an injury and are a natural consequence of the wound healing process. Hypertrophic scars and keloids are types of skin scars that can be caused by a variety of factors, such as pathological fibrosis, inadequate wound closure, extracellular matrix (ECM) overproduction, chronic inflammation, and excessive collagen deposition [71,72,73]. MSC derivatives such as exosomes have shown similar therapeutic potential to MSC, and their shuttling miRNAs have beneficial paracrine effects on mitigating scar formation by regulating the proliferation, migration, and myofibroblast transdifferentiation of hypertrophic scar fibroblasts, reducing collagen deposition, inhibiting ECM fibrosis, and regulating the expression of scar-related proteins (Figure 2).

Myofibroblast aggregation is a key factor involved in scar formation, and the induction of TGF-β by miRNAs is closely related to the differentiation of myofibroblasts. For example, blocking the TGF-β2/Smad2 pathway is a way to effectively prevent myofibroblast aggregation and reduce scar formation in vitro and in vivo [74]. MiR-141-3p delivered from ADSC exosomes has been shown to possess important functions in treating hypertrophic scars by inhibiting the proliferation, migration, and myofibroblast transdifferentiation of hypertrophic scar fibroblasts in vitro through targeting TGF-β2 and inhibiting the TGF-β2/Smad pathway [51]. Similarly, UCMSC-exosome-enriched specific miRNAs (miR-21, miR-23a, miR-125b, miR-145) mediate the anti-scarring effect of UCMSC in vitro and in vivo by inhibiting the TGF-β2/Smad2 pathway during the wound healing process [25]. Exosomal miR-29a produced from miR-29a-modified human adipose-derived mesenchymal stem cells has also been shown to have the same effects as miRNAs described above in reducing the formation of pathological scars. Targeting the similar TGF-β2/Smad3 signaling pathway, miR-29a reduces the formation of scars by inhibiting collagen deposition and ECM fibrosis and preventing the migration and proliferation of hypertrophic scar fibroblasts [24]. Intriguingly, one study has reported that a magnetic Fe_3_O_4_ nanoparticle and a static magnetic field can be utilized to stimulate BMSC to secrete exosomes enriched with miR-21-5p, which enhances wound healing with a higher wound closure rate and less scar formation in rats. Exosomal miR-21-5p has been shown to play a crucial role in these effects by inhibiting *SPRY2* and activating the PI3K/AKT and ERK1/2 signaling pathways [54]. Sirtuin1, a class III histone deacetylase, is considered an ideal target for scar prevention and treatment [75]. A study found that ADSC exosomes can also reduce scarring by modulating the miR-181a/sirtuin1 axis [52]. Silent information regulator 1 (SIRT1) is a NAD (+)-dependent protein-modifying enzyme, which can up-regulate the expression of scar-related molecules such as NF-κB, α-smooth muscle actin (α-SMA) and TGF-β1 in HSF. MiR-138-5P in MSC-Exos can directly target SIRT1 by binding to the 3′-UTR of SIRT1, which can inhibit the proliferation, migration, and protein expression of hypertrophic scar fibroblasts and thus alleviate pathological scars [30].

A hypertrophic scar is a fibroproliferative disorder of the dermis after a severe burn injury, which often results in aesthetic disfigurement and dysfunction in patients. Studies have shown that ADSC-Exos can modulate skin fibrosis and have a specific effect on hypertrophic scars. ADSC-Exos can effectively inhibit the proliferation and migration of hypertrophic scar fibroblasts and alleviate fibrosis. Further research has found that the antifibrotic effect of ADSC-Exos-derived miR-192-5P on hypertrophic scar fibroblasts is closely related to inrleukin-17 receptor A (IL-17RA), and the Smad signaling pathway is associated with almost all fibrotic diseases. Li et al.’s work showed that miR-192-5p in ADSC-Exos can reduce HSF fibrosis and specifically target IL-17RA to alter the Smad pathway in proliferative scar formation, thereby speeding up wound healing and decreasing collagen synthesis following severe burn injury [53].

### 2.3. Anti-Aging

The process of biological aging is an inherent attribute of living organisms and is influenced by genetics, but it is also influenced by environmental factors and time to a greater extent [76]. When there is excessive intracellular or extracellular stress or injury, the cell cycle is irreversibly arrested, resulting in cellular senescence [76]. The G1 phase persistent arrest of the cell cycle and the inflammatory response known as the senescence-associated secretory phenotype (SASP) are the hallmarks of aging [8,76]. The acquisition of SASP occurs by activating the SA secretome and altering the milieu surrounding senescent cells through the DNA damage response (DDR) [77,78]. SASP mainly works by attracting phagocytic immune cells and eliminating acutely senescent cells, then replacing them with newly dividing cells [76,79]. However, the acquisition of SASP also results in the activation of DDR and SASP in nearby cells, chronic inflammation, and normal cellular senescence, which lowers immune function and creates a pro-inflammatory environment that first occurs locally and then spreads to the systemic level, accelerating the aging of the body [78,80].

**Figure 3:**
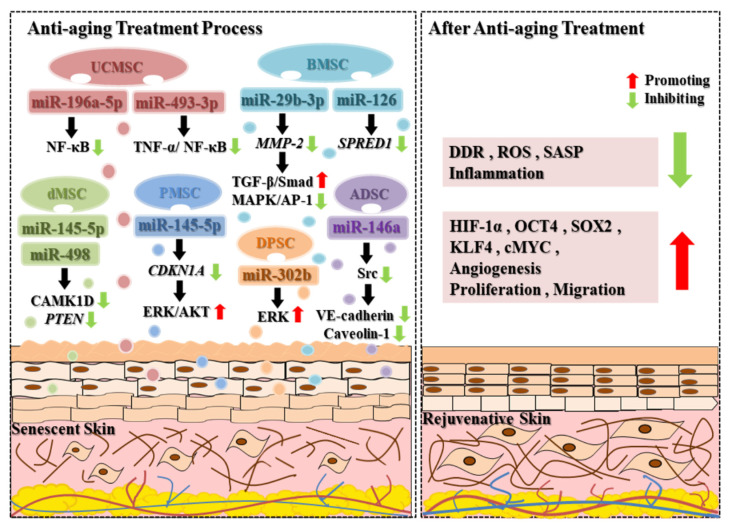
Anti-aging mechanisms of exosomal miRNAs from different sources of mesenchymal stem cells. Exosomes derived from different sources of mesenchymal stem cells exert their anti-aging effects on senescent skin through different miRNA-mediated targets or signaling pathways. After exosome treatment, senescent skin can be rejuvenated by reducing the levels of the DNA damage response (DDR), reactive oxygen species (ROS), the senescence-associated secretory phenotype (SASP), and inflammation, as well as promoting cell proliferation, migration, angiogenesis, and the release of associated factors.

While aging in the skin is perhaps the most manifest among other tissues, it is potentially also one of the most amenable targets for both prevention and rejuvenation due to its prominent location on the surface. The preferred rejuvenation measure among numerous ways would be to reactivate stem cells within the skin tissue, which would reverse the exhaustion of stem cells. Indeed, it has been confirmed that the paracrine effects of MSC are linked to their immunomodulatory properties and strong regenerative capacity, which make them even more effective than stem cell therapy [61,81]. As a result, increasing evidence has shown that extracellular vesicles or exosomes are a new component of SASP and age-related disease markers [82]. In recent years, extracellular vesicles derived from mesenchymal stem cells (MSC-EVs), particularly miRNAs in MSC-EVs, have been extensively studied for skin anti-aging or skin rejuvenation treatment (Figure 3).

One study reported that miR-145-5p loaded on placental-mesenchymal-stem-cell-derived extracellular vesicles (PMSC-EVs) can inhibit high glucose-induced fibroblast senescence injury by targeting *CDKN1A* and activating the ERK/AKT signaling pathway [55]. Excessive accumulation of reactive oxygen species (ROS) can stimulate acetylated p53 to promote cell senescence and apoptosis [83,84]. In a mouse cell aging model, miR-146a derived from ADSC exosomes has been demonstrated to mitigate oxidative-stress-induced endothelial cell senescence by blocking Src kinase activation and inhibiting downstream targets VE-cadherin and Caveolin-1, thus decreasing the levels of DDR, ROS and SASP, which ultimately can stimulate angiogenesis and promote wound healing [57]. Similarly, in the model of high glucose-induced senescent human dermal fibroblasts (HDF), exosomes derived from human decidua mesenchymal stem cells (dMSC) were efficacious in delaying cellular senescence. The mechanism is to inhibit the expression of calcium/calmodulin-dependent protein kinase 1D (CAMK1D) and *PTEN* gene through the up-regulation of miR-145-5p and miR-498, thereby promoting the proliferation and migration of human dermal fibroblasts and inhibiting their apoptosis [56]. Interestingly, Chen et al. developed a continuous nutrient supply culture (CC) strategy to culture and collect umbilical cord MSC-derived EVs (CC-UCMSC-EVs) and found that CC-UCMSC-EVs reduced the accumulation of inflammatory factors through miR-493-3p and miR-196a-5p-mediated inhibition of the NF-κB signaling pathway, promoted the growth and migration of fibroblasts, and increased the regeneration of collagen fibers through activation of the TGF-β/Smad pathway, which ultimately led to the achievement of anti-photoaging repair efficacy of skin [62].

Bone-marrow-mesenchymal-stem-cells-derived extracellular vesicles (BMSC-EVs) also play an extremely important role in skin anti-aging. For instance, Wang et al. have demonstrated for the first time that miR-126 enriched in BMSC-EVs rejuvenates senescent endothelial progenitor cells (EPCs) and promotes tissue regeneration and angiogenesis by targeting *spred-1* [58]. In addition, exosomal miR-29b-3p shuttled by BMSC-EVs can alleviate UVB-induced photoaging and promote the migration of human dermal fibroblasts (HDF) by targeting matrix metalloproteinase-2 (*MMP-2*) [23]. The activation of TGF-β/Smad signaling pathways and inhibition of MAPK/activator protein-1 (AP-1) signaling pathways may play a vital role in preventing UVB-induced cell senescence [23,59,60]. In another research, exosomal miR-302b derived from dental pulp stem cells (DPSCs) can promote cell rapid proliferation and delay aging by up-regulating HIF-1α and multi-functional factors like octamer binding transcription factor 4 (OCT4), SOX2, KLF4, and cMYC through the ERK pathway, changing the mitochondrial metabolism pathway from oxidative to glycolytic metabolism [61].

In conclusion, MSC-derived exosomes possess anti-aging effects through their unique miRNAs. They have the potential to reduce senescent cells in tissues by inducing proliferation and reducing SASP in senescent cells. It has been reported that removing senescent cells from tissues leads to a pro-regenerative environment [85]. Therefore, using MSC-derived exosomal miRNAs to remove senescent cells could be a more effective method for promoting skin tissue regeneration or rejuvenation.

### 2.4. Hair Loss

It is widely accepted that hair regeneration plays a significant role in the complete regeneration of the skin. Furthermore, hair loss or alopecia is one of the obvious phenotypes associated with aging. Alopecia can be triggered by numerous factors, including aging. Aging-associated alopecia is known to be associated with follicle quiescence and miniaturization [86,87,88]. Hair follicles (HFs) undergo regular cycles of growth, degeneration, and regeneration [89]. HF stem cells found in the dermal papilla at the base of the follicle and the bulge region are responsible for regulating the regenerative phase, which shows a decline in activity as we age [90,91]. Additionally, dermal papilla cells (DPCs) are known to support hair growth and regulate the hair cycle, and the key to regulating hair regeneration is the development of the HF cycle from the resting period to the growth period [92,93].

Stem cells, particularly exosomes derived from stem cells, are now being used to treat hair loss, reverse skin aging, and achieve rejuvenation effects. Stem-cell-derived exosomes play a key role in this process of regulating cellular pathways that are crucial for the growth, differentiation, and function of hair epithelial cells, such as inhibition and activation of BMP/TGF-β and WNT signaling pathways [63,64,93,94,95]. The growth and development of the HF are usually controlled by exosomal miRNAs through intercellular communication [64,93,95]. MSCs have garnered increased attention among all types of stem cells because of their potential to regenerate and repair tissue as well as their application for hair regeneration. In recent years, it has been proven that MSC-derived exosomes transport miRNAs, which can affect hair restoration and prevent hair loss. It has been proven that exosomes from ADSC have been shown to promote the proliferation, migration, and apoptosis remission of DPC, and ADSC exosome therapy has a positive impact on the promotion of hair regeneration by regulating miR-22, Wnt/β-catenin signaling, and TNF-α signaling [63]. MiR-22 is a key regulator of the hair cycle that promotes the transition from anagen to catagen. ADSC-Exos stimulated HF growth by down-regulating miR-22 and activating the Wnt/β-catenin pathway [63]. ADSC-Exos carrying miR-122-5p antagonize the inhibition of dihydrotestosterone (DHT) on HF growth and up-regulate β-catenin and versican expression in vitro and in vivo, restoring HF size and dermal thickness [64]. ADSC-Exos enhance normal HF growth in androgen alopecia (AGA) by activating miR-122-5p and inhibiting the TGF-β1/Smad3 axis [64].

## 3. Improving Exosomal miRNA Content and Function through Bioengineering Approaches

**Figure 4:**
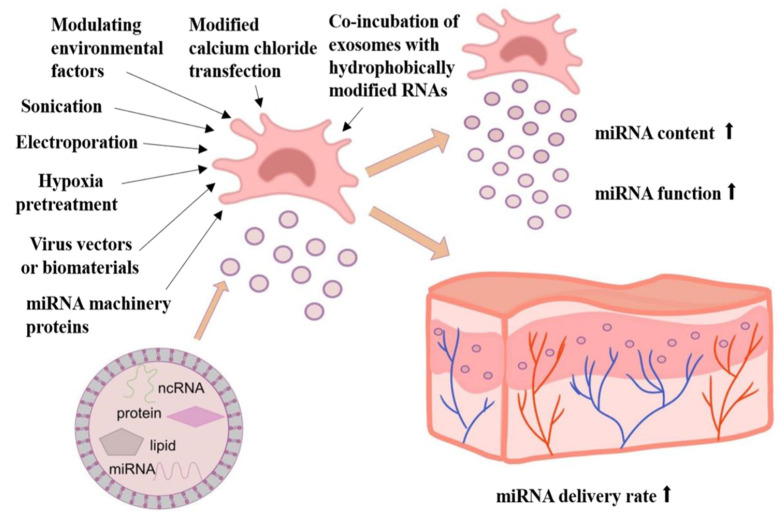
Bioengineering methods to improve exosomal miRNA content and function. A variety of bioengineering approaches can be employed to enhance the content and function of miRNAs secreted by exosomes, such as hypoxic preconditioning of parental cells, modulation of environmental factors, modified calcium chloride transfection methods, electroporation, sonication, co-incubation of exosomes with hydrophobically modified RNAs, and regulation of miRNA machinery proteins. In addition, the use of viruses or biomaterials as vectors can significantly improve the content and function of exosomal miRNAs by increasing their delivery rate.

MSC-derived exosomes have received increasing attention because of their therapeutic potential and lower adverse effects. The nanosize and the surface lipid and protein composition of the exosomes enable them to easily traverse microvessels and other tissues, enabling MSC therapy to overcome its limitations [96]. MiRNA content manipulation in exosomes will be crucial for enhancing future therapeutic applications. Consequently, the isolation, purification, and manipulation of exosome cargo are necessary to expedite the efficacy of miRNA-based therapy and optimize its yield [4]. The content and function of exosomal miRNAs may be related to the type of MSC, the environment, RNA-binding proteins, and other factors [96]. Currently, an enhancement in exosomal miRNA content and function can be achieved through a variety of strategies, such as hypoxia pretreatment of parental cells, using virus vectors or biomaterials as carriers, manipulating environmental factors, and other miRNA loading or transfection methods mediated by physical or chemical means (Figure 4).

### 3.1. Hypoxia-Induced MSCs Produce Wound-Protective Exosome miRNAs

Hypoxic environment is a very common condition during skin wounds, inflammation, and infection. However, hypoxia is a vital regulator of gene expression in an early wound environment. It has been shown that MSC-Exos derived from hypoxia pretreatment are capable of up-regulating miRNA content in exosomes, and MSC-Exos for skin belly regeneration are more effective in a hypoxic environment compared with MSC-Exos in a normal oxygen environment [50]. It has been demonstrated that hypoxia can enhance the exosome-mediated paracrine effects of UCMSC on endothelial cell proliferation and migration and then induce the transcription of miR-125b in UCMSC. Exosomal miR-125b activated in the hypoxic microenvironment can target and inhibit the expression of *TP53INP1*, a protein with multiple functions involved in cell cycle arrest and apoptosis, implying that the regulation of exosomal miR-125b/*TP53INP1* signaling is crucial for accelerating skin wound repair by promoting cell growth and migration and decreasing cell apoptosis [50].

### 3.2. Improving the miRNA Content and Function by Using Virus Vectors or Biomaterials as Carriers

Pretreatment of MSC can directly increase the level of miRNAs in MSC-derived secretions. And beyond that, the transduction of MSC with lentivirus is another method for up-regulating the content of miRNAs in secretions. However, virus vectors usually have the disadvantages of potential mutations, toxicity, and production difficulties [31]. In addition to the conventional virus vectors, there are also materials such as liposomes and nanoparticles that can act as carriers and possess less toxicity and greater effectiveness [31].

Up-regulation of miR-223 has been shown to drive macrophage polarization toward the anti-inflammatory M2 phenotype, which may contribute to accelerating wound healing [97]. Saleh and co-workers used hyaluronic acid nanoparticles as a vehicle to deliver miR-223 to wound sites, resulting in a successful inhibition of the inflammatory response and faster wound healing [97]. One study reported that silk fibroin is a biocompatible polymer that can be made into nanostructures called nanosilks. Nanosilks are characterized by high intensity density ratios and exhibit strain hardening. They have previously produced free radical-scavenging cerium oxide nanoparticles (CNPs) conjugated to anti-inflammatory miRNAs (CNP-miR-146a), which can shorten wound healing time after treatment and can be efficiently delivered to the wound via nanofilament solution to promote wound closure in diabetic mice [98,99]. A study has proven that exosomes derived from miR-126-3p overexpressed synovial mesenchymal stem cells (SMSCs) are encapsulated in hydroxyapatite-chitosan composite hydrogels as a wound dressing, which possesses the property of longer controlled release and successfully promotes wound surface re-epithelialization, accelerates angiogenesis, and expedites collagen maturity by activating PI3K/AKT and MAPK/ERK pathways for the healing of diabetic chronic wounds [41]. In another study, bilayered thiolated alginate/PEG diacrylate hydrogels were used as a controlled release carrier of BMSC-EVs to achieve rapid and scarless wound healing; BMSC-EVs containing enriched miR-29b-3p were released from the upper layer of the hydrogels and suppressed excessive angiogenesis and collagen deposition during the late proliferation and maturation phases, thus reducing the formation of hypertrophic scars and accelerating scarless wound healing [100].

### 3.3. Modulative Environmental Factors Regulate Exosomal miRNA Content and Function

The composition of exosomes is closely related to the growth environment from which MSCs are derived, and several studies have implemented techniques to manipulate environmental factors, such as the fact that exosomes derived from MSC grown in 3D culture conditions contain more contents like miRNAs and are more effective in promoting functional recovery. Another study reported that exosomes released from placenta-derived mesenchymal stem cells (PMSCs) with nitric oxide (NO) stimulation revealed superior angiogenic effects as a result of the enhanced expression of proangiogenic molecules such as miR-126 in the exosomes, implying that NO has the potential to enhance the proangiogenic compositions of exosomes and their proangiogenic capacity [101]. Wound healing can be enhanced by both BMSC-Exos and magnetic nanoparticles. In Wu et al.’s work, bone mesenchymal stem cells were stimulated with a magnetic Fe3O4 nanoparticle and a static magnetic field to create novel exosomes. These exosomes improved angiogenesis and fibroblast function, speeding up wound closure, and the up-regulation of miR-21-5p in these exosomes may have been a contributing factor [54]. Yang et al. utilized blue light (455 nm) to illuminate UCMSC to produce exosomes with improved therapeutic efficacy by elevating levels of miR-135b-5p and miR-499a-3p in exosomes to enhance proangiogenic capacity [102].

### 3.4. Other Methods to Improve the miRNA Content of Exosomes

Exosomes contain a lipid membrane bilayer structure, which makes it possible for hydrophobic compounds to passively enter through direct incubation [103]. For example, Sun et al. utilized hydrophobic binding to load the hydrophobic drug curcumin into mouse lymphoma EL-4 exosomes through direct incubation for anti-inflammatory and antioxidant treatment without causing any damage to the membrane integrity of the exosomes [104,105]. However, RNA is unable to passively diffuse across the hydrophobic exosome lipid bilayer membrane during incubation due to its hydrophilic nature [104]. Therefore, hydrophobic modification of RNAs is required for easy entry into exosomes through co-incubation. For instance, let-7b miRNAs that have been hydrophobically modified down-regulate their oncogene target high mobility group AT-hook 2 (*HMGA2*) in vivo and increase their biodistribution to non-small-cell lung cancer after delivery [106]. The data indicate that the cholesterol fraction is coupled to siRNA and stabilized with complete phosphorothioate tails in order to modify the siRNA to have higher hydrophobic properties, which enables effective and steady loading of siRNA onto the exosome via direct co-incubation without destroying the entire vesicle [107].

In addition to the hydrophobic binding methods described above, membrane permeabilization strategies, including electroporation, sonication, and other methods, have also been developed for loading exosome cargo [104].

Electroporation is an effective tool for performing gene transfection and introducing exogenous RNAs into exosomes, or MSC, and electrical impulses in microseconds to milliseconds are utilized to cause a temporary decrease in the stability of MSC and exosome membranes, enabling cargo to enter cells or exosomes [108]. For example, miR-17-92 can be loaded into MSC through electroporation to enhance their nerve protection function [96]. Xiong and his colleagues loaded miR-542-3p into BMSC-Exos using electroporation, which strengthened the proliferation, migration, and angiogenesis of HSF and HMEC in vitro and expedited wound closure [109]. Ultrasound is also a method of loading miRNAs by applying a low sound frequency to destroy the integrity of the exosome membrane and transfer RNAs to the exosomes, which has been confirmed to significantly raise the miRNA content of exosomes [96]. There is also a modified method for transfection using calcium chloride, which includes a slow mixture of phosphate-buffered saline and CaCl in two solutions containing the desired small RNA and a heat shock step to directly load miRNA mimics or inhibitors into isolated exosomes to promote the miRNA delivery process [96].

Furthermore, miRNA machinery proteins play a significant role in regulating miRNA content. For instance, the Argonaut-2 protein (Ago2) and Dicer are two important miRNA effectors [110,111]. It has been proven that Ago2 and Dicer can enrich miRNAs to regulate local axon outgrowth; knocking down the Ago2 in BMSC can attenuate the effects of BMSC-EVs and decrease the content of miRNAs [110,111,112].

## 4. Conclusions and Prospect

The skin is the largest organ in the human body, the initial protective barrier, and our most crucial organ for protecting ourselves from infection and harm. Despite the fact that most skin-related diseases are usually not life-threatening, they can have a significant impact on an individual’s quality of life, mental health, and health care budgets [3,4,5,6]. Skin regeneration and rejuvenation are aimed at restoring the structure and the function of skin, reducing scar formation, improving the quality and effectiveness of damaged skin, and counteracting age-related morphogenetic changes [113,114].

Skin rejuvenation and the repair of damaged skin remain a major challenge in modern medicine. As previously mentioned, the ability of MSC to induce anti-inflammatory conditions and promote angiogenesis, as well as other promising properties, highlights the infinite potential and importance of MSC in the field of skin regeneration [7]. However, the use of MSC-based exosome therapy has made a significant contribution to the advancement of skin regeneration and rejuvenation, which offers a more feasible approach for bench-to-bedside translation compared to cell-based therapy. Despite the fact that MSC has assumed an important role in clinical research in this area of skin rejuvenation, MSC-Exos therapy still poses considerable challenges to be overcome before it can enter clinical practice. MSC-Exos, as a cell-free therapy, have significant advantages over MSC treatment due to their stability and ability to overcome various safety concerns such as oncogenesis, immunogenicity, and genomic mutations [7,8,15]. Adipose stem cell exosomes, for instance, work better to cure acne scars when combined with fractional carbon dioxide laser, according to a clinical investigation. The study also showed that an adjuvant treatment combined with ADSC-Exos possesses clinical efficacy and safety in acne scarring applications [115]. Similarly, exosomes from ADSC were used to effectively and safely treat aging facial skin through a combination of treatments with microneedling. This combination greatly promoted facial skin recovery [116]. Therefore, we consider that a possible therapeutic technique to stimulate skin regeneration is to combine MSC exosomes with traditional methods.

New studies have demonstrated the potential of MSC-Exos in skin therapy. MSC-based exosome therapy has opened the door to innovative approaches for repairing and regenerating damaged skin, as well as preventing and reversing skin aging. The present understanding is that the function of exosomes is determined by their contents. Moreover, exosome content can vary due to a number of factors, including tissue source, cell type, donor characteristics, and isolation methods. It is therefore necessary to standardize exosome contents to ensure that they contain consistent and reliable components, regardless of their source. The variability in exosome content can be controlled by establishing standardized protocols for isolation, purification, and characterization and ensuring the survival of specific bioactive molecules. MiRNA is one of the contents of exosomes and plays an important role in the regulation of exosome function. MSC can release miRNAs in exosomes, which can be taken up by certain recipient cells and induce phenotypic and functional changes [14,20]. MiRNAs are small molecules that can target multiple genes simultaneously, are relatively stable, and are suitable for technical manipulation [20,21,22]. Despite the advantages and benefits of MSC-based exosome therapy, there are still obstacles such as low production yield, low miRNA content in exosomes, and functional heterogeneity derived from different MSC sources. Thus, increasing attention has been focused on controlling the miRNA content secreted by the exosome, and there are various bioengineering methods available for isolation, purification, and manipulation of exosome release. However, the variety of miRNA sources and modes of action may produce different results for different or the same wound types. As a result, it is necessary to further deepen the exploration of miRNA delivery time and delivery route. To evaluate miRNA expression accurately, all factors must be taken into account, and the miRNA analysis method must be improved for clinical application. It is important to note that there are still many outstanding issues regarding the clinical application of exosomes, such as the mechanism of activity, effectiveness, and safety of MSC-Exos. Therefore, in order to overcome these challenges, efforts should be made to develop standardized guidelines and quality control measures for exosome production.

Meanwhile, there is still a need for an in-depth understanding of the potential mechanisms of exosomal miRNAs of MSC origin in dermal therapy and to conduct extensive clinical trials to ensure the efficacy and safety of any therapeutic interventions, especially in the case of topical applications. The development of molecular-based medicines and novel material carriers opens up vast opportunities for more effective and precise exosomal miRNA-based skin therapies. However, the market availability of any therapeutic agent is potentially risky until clinical studies are completed. In conclusion, MSC exosomal miRNAs act as skin boosters to delay skin aging, promote skin regeneration, and maintain skin health, providing a novel therapeutic approach to the diagnosis of skin damage and aging. 

## Figures and Tables

**Table 1:** Exosomal miRNAs derived from mesenchymal stem cells involved in the process of skin regeneration and rejuvenation.

miRNA	Source of MSC	Targets or Pathways	Processes and Effects	References
Wound healing				
miR-181c	UCMSC	TLR4; NF-κB/P65	lnflammatory cytokine production ↓	[32]
miR-146a	MSC	*IRAK1*, *TRAF6*, *NF-κB*	lnflammatory cytokine production ↓; Inflammatory gene expression ↓	[33,34]
miR-223	BMSC	Pknox1	M2-phenotype macrophage polarization ↑	[35]
miR-let-7b	UCMSC	*TLR4*/NF-κB/ STAT3/AKT	M2-phenotype macrophage polarization ↑; lnflammatory cytokine production ↓	[36]
miR-34a-5p miR-124-3p miR-146a-5p	ADSC	*ARG1*, *CD206*, *TSG-6*, *TGF-β1*	M2-phenotype macrophage polarization ↑	[37]
miR-146a miR-132	ADSC	*ROCK1*/*PTEN*	Pro-angiogenic gene expression ↑; Proliferation and tube formation of HUVEC ↑	[38]
miR-17-5p	UCMSC	AKT/HIF-1α/ VEGF	Proliferation, migration, and tube formation of HUVEC ↑	[26]
miR-221-3p	BMSC	AKT/eNOS	Proliferation, migration, and tube formation of HUVEC ↑; VEGF secretion ↑; Granulation tissue formation ↑	[39]
miR-126	BMSC	*PIK3R2*; PI3K/AKT	Proliferation, migration, and tube formation of HUVEC ↑	[40]
miR-126-3p	SMSC	MAPK/ERK; PI3K/AKT	Migration of HMEC ↑; Capillary-network formation ↑	[41]
	BMSC	*SPRED1*/Ras/ERK	Proliferation, migration, and tube formation of HUVEC ↑	[42]
miR-125a	ADSC	*DLL4*	Endothelial tip cell formation ↑	[43]
miR-486-5p	ADSC	*Sp5*/CCND2	Proliferation and migration of HSF and HMEC ↑; HMEC angiogenesis ↑	[44]
miR-19b↓	ADSC	H19/*SOX9*/ Wnt/β-catenin	Proliferation, migration and invasion of HSF ↑; Collagen fibre formation ↑	[45]
miR-19b	ADSC	*CCL1*/TGF-β	Proliferation and migration of HSF and HaCaT cells ↑; HSF, HaCaT and endothelial cell apoptosis ↓	[46]
miR-135a	AMSC	*LATS2*	Proliferation and migration of fibroblasts ↑	[28]
miR-93-3p	BMSC	APAF1	Proliferation and migration of HaCaT cells ↑; Cellular apoptosis ↓	[27]
miR-150-5p	MSC	*PTEN*	Proliferation and migration of HaCaT cells ↑	[47]
miR-27b	MSC	ITCH	Proliferation and migration of HSF and HaCaT cells ↑; Collagen fiber proliferation ↑; Epithelialization ↑	[48]
miR-10b	ADSC	PEA15	Proliferation and migration of HaCaT cells ↑; Cellular apoptosis ↓	[49]
miR-125b	UCMSC	*TP53INP1*	Proliferation and migration of endothelial cells ↑; Cellular apoptosis ↓	[50]
Skin scar				
miR-141-3p	ADSC	TGF-β2/Smad2/3	Proliferation and migration of hypertrophic scar fibroblasts ↓; Myofibroblast transdifferentiation ↓	[51]
miR-21, miR-23a, miR-125b, miR-145	UCMSC	TGF-β2/Smad2	Myofibroblast transdifferentiation ↓; Collagen deposition ↓	[25]
miR-29a	ADSC	TGF-β2/Smad3	Migrating and proliferating of hypertrophic scar Fibroblasts ↓; Collagen deposition and ECM fibrosis ↓	[24]
miR-181a	ADSC	Sirtuin1	Myofibroblast transdifferentiation ↓; Collagen deposition ↓	[52]
miR-138-5P	MSC	SIRT1	Proliferation, migration and protein expression in human skin fibroblasts ↓	[30]
miR-192-5P	ADSC	IL-17RA/Smad	Proliferation and migration of hypertrophic scar fibroblasts ↓; Myofibroblast transdifferentiation ↓; Collagen deposition ↓	[53]
miR-21-5p	BMSC	*SPRY2*	Migrating and proliferating of hypertrophic scar fibroblasts ↓; Collagen deposition ↓	[54]
Anti-aging				
miR-145-5p	PMSC	*CDKN1A*; ERK/AKT	Proliferation and migration of senescent fibroblasts ↑; Cellular senescence ↓; Cellular apoptosis ↓	[55]
	dMSC	CAMK1D; *PTEN*	Proliferation and migration of senescent dermal fibroblasts↑; Cellular senescence ↓; Cellular apoptosis ↓	[56]
miR-146a	ADSC	Src kinase; VE-cadherin; Caveolin-1	Angiogenesis ↑; Cellular senescence ↓; Migration of senescent endothelial cells ↑	[57]
miR-126	BMSC	*Spred-1*	Angiogenesis ↑; Tissue regeneration ↑	[58]
miR-29b-3p	BMSC	*MMP-2*; TGF-β/Smad; MAPK/AP-1	Migration of human dermal fibroblasts ↑; Photoaging ↓; Matrix metalloproteinase ↓; Procollagen ↑	[23,59,60]
miR-302b	DPSC	ERK	Cell proliferation ↑; Stemness ↑; Cellular senescence ↓	[61]
miR-498	dMSC	CAMK1D; *PTEN*	Proliferation and migration of senescent dermal fibroblasts ↑; Cellular senescence ↓; Cellular apoptosis ↓	[56]
miR-493-3p	UCMSC	TNF-α/NF-κB	Growth and migration of fibroblasts ↑; Procollagen ↑; Oxidative stress levels ↓; Cellular senescence ↓	[62]
miR-196a-5p	UCMSC	NF-κB		
Hair loss				
miR-22 ↓	ADSC	Wnt/β-catenin; TNF-α	Hair growth ↑; Hair regeneration ↑; Dermal thickness ↑; Proliferation and migration of DPC ↑; Anti-apoptosis ↑	[63]
miR-122-5p	ADSC	TGF-β1/Smad3	Hair bulb size ↑; Dermal thickness ↑	[64]

Note: ↑: Promoting; ↓: Inhibiting.

## Data Availability

Not applicable.

## Abbreviations

The following abbreviations are used in this manuscript:

MSC	Mesenchymal stem cells
EVs	Extracellular vesicles
mRNAs	Messenger RNAs
miRNAs	MicroRNAs
Exos	Exosomes
MVs	Microvesicles
MSC-Exos	Mesenchymal stem cells-derived exosomes
lncRNAs	Long non-coding RNAs
UCMSC	Umbilical cord mesenchymal stem cells
UCMSC-Exos	Umbilical cord mesenchymal stem cells-derived exosomes
NF-κB (*NF-κB*)	Nuclear transcription factor-kappa B
TLR	Toll-like receptor
IL-1b	Interleukin-1b
TNF-α	Tumor necrosis factor-α
*IRAK1*	IL-1 receptor-associated kinase 1
*TRAF6*	TNF receptor-associated factor 6
BMSC	Bone marrow mesenchymal stem cells
ADSC	Adipose mesenchymal stem cells
*ARG1*	Arginase 1
*TSG-6*	Tumor necrosis factor-inducible gene 6
TGF-β1(*TGF-β1*)	Transforming growth factor beta 1
IFN-γ	Interferon-γ
VEGF	Vascular endothelial growth factor
MAPK	Mitogen-activated protein kinase
M1/2	Macrophage1/2
LPS	Lipopolysaccharide
AKT	Serine/threonine-protein kinase B
PI3K	Phosphatidylinositol 3-kinase
HIF-1α	Hypoxia inducible factor-1 alpha
eNOS	Endothelial nitric oxide synthase
*PIK3R2*	Phosphoinositol-3 kinase regulatory subunit 2
HUVEC	Human umbilical vein endothelial cells
ERK	Extracellular signal-related kinases
*SPRED1*	Sprouty-related EVH1 domain-containing protein 1
*DLL4*	Delta-like 4
*ANGPT1*	Angiopoietin1
*KDR*	Kinase insert domain receptor
*THBS1*	Thrombospondin-1
*VASH1*	Vasohibin-1
CCND2	Cyclin D2
HMEC	Human dermal microvascular endothelial cells
HaCaT	Human keratinocytes
APAF1	Apoptotic protease-activating factor 1
PTEN(*PTEN*)	Phosphatase and tensin homolog
CDK6	Cyclin dependent kinase 6
HSF	Human skin fibroblasts
ECM	Extracellular matrix
SIRT1	Silent information regulator 1
IL-17RA	Interleukin-17 receptor A
STAT3	Signal transducer and activator of transcription 3
PDGF	Platelet-derived growth factor
*LATS2*	Large tumor suppressor kinase 2
H19	lncRNA H19
ADSC-Exos	Adipose mesenchymal stem cells-derived exosomes
ITCH	Itchy E3 ubiquitin protein ligase
SRY	Sex-determining region Y
*SOX9*	SRY-related high-mobility-group box 9
CCL1	Chemokine CC motif ligand 1
*TP53INP1*	Tumor protein p53 inducible nuclear protein 1
α-SMA	α-smooth muscle actin
SASP	Senescence-associated secretory phenotype
DDR	DNA damage response
MSC-EVs	Mesenchymal stem cells-derived extracellular vesicles
PMSC-EVs	Placental mesenchymal stem cells-derived extracellular vesicles
ROS	Reactive oxygen species
BMSC-EVs	Bone marrow mesenchymal stem cells-derived extracellular vesicles
EPC	Endothelial progenitor cells
HDF	Human dermal fibroblasts
*MMP-2*	Matrix metalloproteinase-2
AP-1	Activator protein-1
DPSC	Dental pulp stem cells
UVB	Ultraviolet B
OCT4	Octamer binding transcription factor 4
DPC	Dermal papilla cells
HF	Hair follicles
DHT	Dihydrotestosterone
AGA	Androgen alopecia
CNP	Cerium oxide nanoparticles
SMSC	Synovial mesenchymal stem cells
PMSC	Placenta-derived mesenchymal stem cells
NO	Nitric oxide
*HMGA2*	High mobility group AT-hook 2
Ago2	Argonaut-2 protein
AMSC	Amniotic mesenchymal stem cells
dMSC	Decidua mesenchymal stem cells
*ROCK1*	Rho associated coiled-coil containing protein kinase 1
